# Experimental and *In-Silico* Investigation of Anti-Microbial Activity of 1-Chloro-2-Isocyanatoethane Derivatives of Thiomorpholine, Piperazine and Morpholine

**DOI:** 10.1371/journal.pone.0170150

**Published:** 2017-01-20

**Authors:** Charles O. Nwuche, Oguejiofo T. Ujam, Akachukwu Ibezim, Ifeoma B. Ujam

**Affiliations:** 1 Department of Microbiology, University of Nigeria, Nsukka, Enugu State, Nigeria; 2 Japan International Research Center for Agricultural Sciences (JIRCAS), Tsukuba, Ibaraki, Japan; 3 Department of Pure and Industrial Chemistry, University of Nigeria, Nsukka, Enugu State, Nigeria; 4 Department of Pharmaceutical and Medicinal Chemistry, University of Nigeria, Nsukka, Enugu State, Nigeria; 5 Department of Pharmacognosy, University of Nigeria, Nsukka, Enugu State, Nigeria; Aligarh Muslim University, INDIA

## Abstract

The Antibiogram properties of 1-chloro-2-isocyanatoethane derivatives of thiomorpholine (CTC), piperazine (CPC) and morpholine (CMC) were evaluated by the approved agar well diffusion, the minimum inhibitory concentration (MIC) and in silico techniques. A total of fourteen microbial cultures consisting of ten bacteria and four yeast strains were used in the biological study while affinity of the compounds for DNA gyrase, a validated antibacterial drug target, was investigated by docking method. Results indicate that both thiomorpholine and piperazine had zero activity against the Gram negative organisms tested. With morpholine, similar result was obtained except that cultures of Escherichia coli (ATCC 15442) and Salmonella typhi (ATCC 6539) presented with weak sensitivity (7–8 mm) as shown by the inhibition zone diameter (IZD) measurement. The Gram positive organisms were more sensitive to morpholine than the other compounds. The highest IZD values of 15–18 mm were achieved except for Streptococcus pneumoniae (ATCC 49619) in which mobility of the compound stopped after 12 mm. S. pneumoniae was resistant to both thiomorpholine and piperazine. The yeast strains were not sensitive to any of the studied compounds investigated. The MIC tests evaluated against a reference antibiotic show that while morpholine was most active at 4 μg.ml-1 against both *B*. *cereus* ATCC (14579) and *B*. *subtilis*, the least active compound was thiomorpholine which inhibited *S*. *aureus* (ATCC 25923) at 64 μg.ml-1. The three compounds demonstrated high affinity for the target protein (DNA gyrase) ranging from -4.63 to -5.64 Kcal/mol and even showed better ligand efficiencies than three known antibiotics; chlorobiocin, ciprofloxacin and tetracycline. This study identified the studied compounds as potential antibiotic leads with acceptable physicochemical properties and gave the molecular basis for the observed interactions between the compounds and the target protein which can be harnessed in structural optimization process.

## Introduction

There has been increased interest in antimicrobial activity of compounds derived from multifunctional heterocyclic molecules especially those from thiomorpholine [1–3] and piperazine [4–6] and morpholine [7–9]. This is partly because they are privileged molecules for the preparation of bioactive compounds but mainly because they offer better solubility and pharmacokinetics [10–13]. Compounds of these moieties are known to be bioactive across a number of different therapeutic areas [14,8,2]. Therefore, there is a big scope in inventing novel antimicrobial agents with potential anti-pathogenic activities base on these heterocyclic compounds toward ameliorating the problem of antimicrobial drug resistance. Potentially, exploiting the synthetic scaffold offered by morpholine, thiomorpholine and piperazine through the heterocyclic amine functional group results in more functionalized derivatives that exhibit interesting anti-pathogen activities.

The advent of antibiotics in the treatment of bacterial infections had a defining revolutionary impact in modern medicine. Today, very many diseases which were once major scourges of mankind have literally been put under control if not completely eradicated. However, not long ago, reports began to emerge of many disease pathogens which had acquired resistance to many antibiotics previously used against the invading organisms [15]. It is believed that the effectiveness and easy access of the early antimicrobials was responsible for the widespread problems of antibiotic resistance encountered today. Drug resistance is a serious challenge currently plaguing the global health community and threatens to obliterate the many health benefits the antibiotic era had achieved. Clearly, the present situation looks dire and portends immense danger to the continued human existence except adequate measures are effectively mobilized. Antibiotic resistance results from drug overuse especially in animal husbandry [16–18] and prophylaxis [19], inappropriate (incorrect or suboptimal) application particularly during self-prescription and medication as well as widespread usage in hospitals [20]. These avenues promote the selection of bacterial strains that no longer respond to treatment [21,22]. During antibiotic therapy, best fitted and genetically enhanced bacterial strains are preferentially selected due to their ability to survive high doses of the antibiotic [23,24]. These resistant strains flourish afterwards while the susceptible strains are wiped out by the effects of the drug. Therefore the key to combating the enormous challenge posed by antibiotic resistance in the healthcare industry is in the continuing search for new antibiotics and antimicrobials to replace the continuously increasing numbers of ineffective and decommissioned ones [25–27]. This is imperative to the overall sustenance of the global healthcare structure. In order to contribute to the ongoing research in this field, a report on the effectiveness of three organic compounds, *N*-(2-chloroethyl)thiomorpholine-4-carboxamide (CTC), *N*^1^,*N*^4^-bis(2-chloro-ethyl)piperazine-1,4-dicarboxamide (CPC), *N*-(2-chloroethyl)morpholine-4-carboxamide (CMC) evaluated against different classes of microorganisms is presented. In addition rational structural optimization of potential drug molecules is performed following the guidance of their intermolecular interaction with a specific drug target [28]. Therefore, the antibiotic potency of the compounds was assessed, *in-silico*, based on their ability to bind to DNA gyrase isolated from *E*. *coli*. Also oral bioavailability profile of the compounds was evaluated using selected molecular descriptors to avoid potential waste of resources on molecules that could pose pharmacokinetic challenge.

## Materials and Methods

1-chloro-2-isocyanatoethane, thiomopholine, morpholine and piperazine were supplied by Sigma-Aldrich. Laboratory regent grade diethyl ether (EMD Chemicals) was used as the reaction solvent without further purification. ClCH_2_CH_2_NHC(O)N(CH_2_CH_2_)_2_S [29] (CTC), ClCH_2_CH_2_NHC(O)N(CH_2_CH_2_)_2_NC(O)NHCH_2_CH_2_Cl [30] (CPC) and ClCH_2_CH_2_NHC(O)N (CH_2_CH_2_)_2_O [31], (CMC) were synthesized according to literature procedures.

### Microorganisms and Culture Conditions

Eleven bacteria and four yeast cultures were used in the study. The organisms were obtained from the stock collections of the Department of Microbiology, University of Nigeria, Nsukka and Bioresources Development and Conservation Programme (BDCP), Nsukka, Nigeria. The Gram positive organisms were *Staphylococcus aureus* ATCC 25923, *Bacillus cereus* ATCC 14579, *Streptococcus pneumonia* ATCC 49619, and *Bacillus subtilis*. The Gram negatives were composed of *Escherichia coli* ATCC 15442, *Salmonella enterica* serovar Enteritidis ATCC 13076, *Salmonella enterica serovar* Wangata NCTC 8276, *Salmonella typhi* ATCC 6539 and *Enterococcus faecalis* ATCC 29212. The yeast strains used were *Candida albicans* ATCC 10231, *Saccharomyces cerevisiae*, *Candida utilis* ATCC 22023 and *Candida glabrata* ATCC 90030. *B*. *subtilis* was isolated from agricultural soils. One loopful of culture of each bacteria specie was transferred into nutrient both (NB) (Lab M) and incubated at 37°C for 24 h except for *B*. *cereus* ATCC 14579 and *B*. *subtilis* which were kept at 30°C. The yeast strains were cultured in yeast extract-peptone-dextrose (YPD) broth (Oxoid) at 30°C for 24 h.

### Antimicrobial Susceptibility Testing

The agar well diffusion method of Tag and Mcgiven [32] was used in determining the antimicrobial properties of the synthesized compounds while the minimum inhibitory concentration (MIC) was evaluated by the broth dilution technique approved by the National Committee for Clinical Laboratory Standards (NCCLS) [33].

### Agar Well Diffusion

Fresh stock solutions (1000 μg.mL^-1^) of the synthesized compounds were prepared in dimethylsulfoxide (DMSO). The inoculum suspension of each test bacteria and yeast strains were prepared from overnight broth cultures and the turbidity equivalent adjusted to 0.5 McFarland standard gave a concentration of 1.0 x 10^8^ bacterial cells and 1.0 x 10^6^ yeast cells/ml respectively. In order to determine the antimicrobial activity of each synthesized compound, freshly prepared Mueller Hinton Agar (MHA) plates were seeded with 100 μL broth culture of each bacteria or yeast. Thereafter, 6 mm diameter wells were made on the agar surface with a flamed cork borer before loading the wells with approximately 50 μL of each compound dissolved in DMSO. Plates inoculated with each test bacteria were incubated at 37°C for 24 h with the exception of those containing *B*. *cereus* ATCC 14579 and *B*. *subtilis* which were maintained at 30°C for 24 h. After incubation, the diameters of the inhibition zones formed on the MHA plates were determined (in millimeters) against a metre rule using a pair of dividers. Discs of tetracycline (TE30) were used as positive controls. The measured inhibition zones of the study compounds were compared with those of the reference drugs.

### Minimum Inhibitory Concentration (MIC)

The test bacteria were inoculated into nutrient broth and incubated at 30–37°C for 24 h while yeast were inoculated into yeast extract peptone-Dextrose broth and incubated at 30°C for 48 h. The inoculum was adjusted according to 0.5 McFarland standards. Initially, 100μl of Mueller Hinton Broth (MHB) was placed in each well. After, the compounds were dissolved in DMSO (2 mg.mL^-1^) and transferred into the first well. Two-fold serial dilutions of the compounds were carried out to determine the MIC within the concentration range of 256 to 0.125 μg.mL^-1^. Cultures were grown at 30–37°C for 18–24 h to density of 10^6^ cfu/mL. The lowest concentration of each study compound that resulted in the complete inhibition of the test microorganisms was reported as the MIC (μg.mL^-1^). Similar procedure was performed in the positive controls. Tests were performed in triplicate and results expressed as means.

### Molecular Simulations

The 3-dimensional structures of the three compounds were generated using the graphical user interface implemented in molecular operated environment (MOE) software [34]. The crystal structures of the bacteria DNA gyrase (PDB code: 1KZN) with its cocrystallized inhibitor (chlorobiocin) were downloaded from RCSB protein data bank [35]. The enzyme-inhibitor complexes were prepared according to standard for use in docking calculation [36]. Both the ligands and protein were energy minimized to a gradient of 0.001 kcal/mol using Merck Molecular (MMFF94) Force field [37]. The molecular descriptor calculator included in the QuSAR module of the MOE package was used to calculate the molecular weight (MW), lipophilicity (log *P*), number of hydrogen bond acceptor (HBA) and donor (HBD), number of rotatable bond (NRB), aqueous solubility (log *S*) and total polar surface area (TPSA) of CTC, CPC and CMC. We used AutoDock4.2.0 and Lamarckian Genetic Algorithm for receptor-fixed ligand-flexible docking calculations in this study. The adopted docking protocol was validated using the RMSD method. The final number of grid points in x-, y- and z- axes were 28, 48, 56 and the distance between two connecting grid points was 0.375 Å. All docking and search parameters were set to default. Five GA runs were carried out for each ligand and Discovery Studio Visualizer and PyMol were used to visualize the ligand conformations within the binding cavity of the protein [38–40].

## Results and Discussion

**Fig 1:**
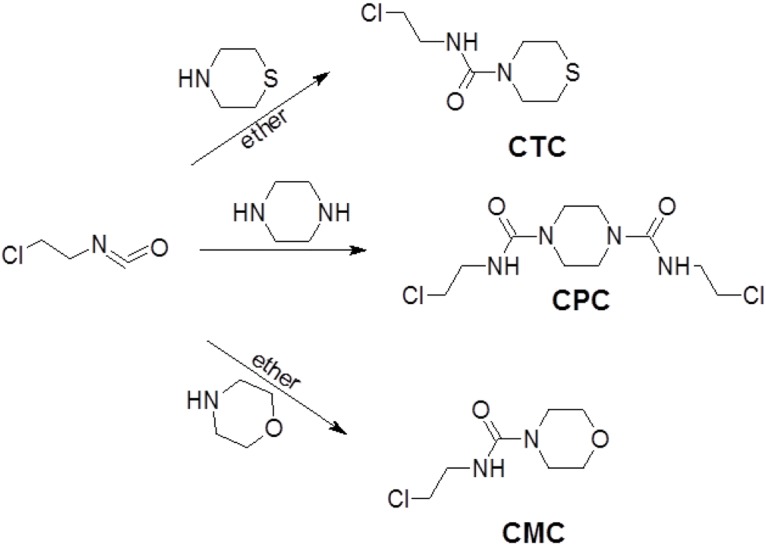
Synthesis of hiomorpholine piperazine and and morpholine derivatives of 1-chloro-2-isocyanatoethane.

The test compound show in Fig 1 were synthesis by the reaction of the heterocyclic compounds thiomorpholine, piperazine, morpholine with 1-chloro-2-isocyanatoethane in diethylether.

### Antimicrobial Tests Results

The results of antimicrobial activities of the study compounds reported as inhibition zone diameter (mm) are presented in Table 1 while the MIC values are shown in Table 2. The test organisms showed varied susceptibility patterns to the study compounds. For instance, CTC and CPC showed zero activity to all the Gram negative organisms tested while CMC presented with negligible antimicrobial effect against *E*. ***c****oli* ATCC 11105 and *S*. *typhi* ATCC 6539. The reason for the apparent antimicrobial resistance of the Gram negative organisms to the test compounds may be connected to the presence of the lipopolysaccharide (LPS) envelop around the cell wall [41]. This layer imposes severe restrictions to the passage of compounds into the cell and is reported to be a critical component of the resistance property of most gram negative organisms to many agents. Several completed studies indicate that drugs must be capable of bypassing this layer and reach intracellular targets before significant bactericidal action could be achieved [41,42]. The lipopolysaccharide layer is absent in Gram positive bacteria and may explain the sensitivity of the test organisms to the study compounds. The MIC profile of the test organisms is presented in Table 2. Of all the organisms studied, only *S*. *pneumoniae* ATCC 49619 was found to be resistant to CTC and CPC.

**Table 1:** Inhibition zone diameter (IZD) of the effect of CTC, CPC and CMC on the test isolates.

Test Microorganism	CTC	CPC	CMC
Gram -ve			
*E*. *coli* (ATCC 11105)	-	-	7
*P*. *aeruginosa* (ATCC 15442)	-	-	-
*S*. *enterica serovar* Enteritidis (ATCC)	-	-	-
*S*. *enterica serovar* Wangata (NTCT)	-	-	-
*S*. *typhii* (ATCC 6539)	-	-	8
*E*. *faecalis* (ATCC 29212)	-	-	-
Gram +ve			
*S*. *aureus* (ATCC 25923)	10	12	15
*B*. *cereus* (ATCC 14579)	13	11	18
*S*. *pneumoniae* (ATCC 49619)	-	-	12
*B*. *subtilis* (Isolated)	12	14	16
Yeast			
*C*. *albicans* (ATCC 10231)	-	-	-
*S*. *cererisiae*	-	-	-
*C*. *utilis* (ATCC 22023)	-	-	-
*C*. *glabrata* (ATCC 90030)	-	-	-

**Table 2:** Minimum inhibitory concentration (MIC, μg.ml^-1^) of the effect of compounds.

Test Microorganisms	CTC	CPC	CMC	Tetracycline
*S*. *aureus* (ATCC 25923)	64	32	8	32
*B*. *cereus* (ATCC 14579)	16	16	4	64
*S*. *pneumoniae* (ATCC 49619)	–	–	32	128
*B*. *subtilis*	32	8	4	64

ND: Not Detected.

The MIC measurement was conducted on the studied compounds that showed significant activity against the test microorganisms as indicated by the IZD data (Table 1). Results show (Table 2) that the MIC of the compounds fell within the 4 to 64 μg.mL^-1^ range. The lowest MIC’s were observed for CMC (4–32 μg.mL^-1^) while the highest which fell within 32–64 μg.mL^-1^ was associated with CTC. The compounds were inactive against *S*. *pneumoniae* except CMC which indicated higher antimicrobial capability than both CTC and CPC. Despite this shortcoming, the results of the present study suggest that the compounds showed promising potential for use as antimicrobial agents against most of the tested Gram positive microorgansims.

### Molecular Modeling

The inhibitory potential of the three compounds (CTC, CPC and CMC) against DNA gyrase was studied based on the fact that topoisomerase II have received the most attention for antibacterial drug discovery [43]. Also, bacterial DNA gyrase is the target of many antibiotics including nalidixic acid, novobiocin, chlorobiocin and ciprofloxacin [44]. Moreover, this enzyme, which causes supercoiling of the DNA by relieving strains while double-stranded DNA is being unwound by helicases, is so essential for bacterial survival but absent from higher eukaryotes [45].

### Docking Calculations

**Fig 2:**
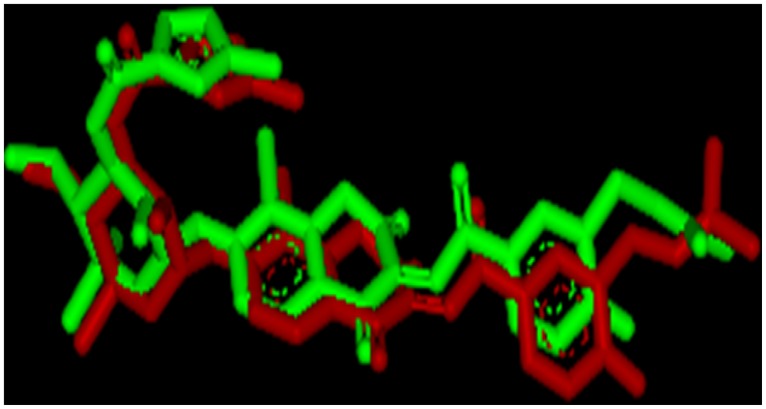
Poses of the x-ray structure of cocrystallized ligand (in red) and the docked ligand (in green).

The docking protocol explained in experimental section gave an rmsd of 2.09 Å which is within the recommended range and hence was implemented in this study (Fig 2) [36].

The docking results were consistent with the biological screening. All the three compounds ranked higher than the cocrystallized inhibitor of DNA gyrase (chlorobiocin) and tetracycline (Table 3).

**Table 3:** *In-silico* inhibitory potential of the three compounds against DNA gyrase.

Compound	ΔG (Kcal/mol)	*K*_i_ (μM)	Ligand efficiency
CTC	-4.81	299.73	0.4
CPC	-5.64	73.47	0.31
CMC	-4.63	404.53	0.39
Chlorobiocin	-4.55	465.26	0.09
Ciprofloxacin	-6.96	7.95	0.29
Tetracycline	-4.64	397.29	0.14

The results of binding free energy present ciprofloxacin as a better inhibitor of DNA gyrase than the studied compounds. However, prioritizing according to their ligand efficiencies shows that CTC, CPC and CMC will more efficiently inhibit the activities of the enzyme than ciprofloxacin and chlorobiocin, two approved drug whose mechanism of actions are by the inhibition of DNA gyrase [46].

### Oral Bioavailability Profile Assessment

Next in our mind was to ascertain how orally bioavailable compounds I-III will be in systemic circulation, should they succeed the rigorous process of clinical trials. The paradigm in drug discovery is the assessment of drug-likeness of potential drug molecule *ab initio* in order not to waste resources on compounds which will pose pharmacokinetic challenge [47]. The widely used Lipinski’s ‘rule of 5’ (ro5) was employed in this exercise. He proposed that a drug which has molecular weight (MW) less than 500, lipophilicity (log *P*) less than 5, number of hydrogen bond acceptor (HBA) and donor (HBD) less than 10 and 5 respectively will be orally bioavailable [48]. In addition, it has been observed that 95% of approved drugs have number of rotatable bond (NRB) of less than 5, an aqueous solubility (log *S*) of more than 5.7 and a total polar surface area (TPSA) of less than or equal to 60 Å^2^ [49]. Analysis of the selected physicochemical properties shown in Table 4, indicates compounds I-III have the requisite pharmacokinetics features to be orally bioavailable. All the compounds respected the ro5. Except for NRB and TPSA where only compound II overshot the criteria for the two properties by very small margin, it was observed that the three compounds, again, had values of NRB, log *S* and TPSA within the recommended range.

**Table 4:** Physicochemical properties used to assess oral bioavailability of drug.

Compounds	NRB	MW	HBA	HBD	lip_violation	log *S*	Log *P*	TPSA
CTC	4	208.71	3	1	0	-1.40	0.14	32.34
CPC	8	297.19	6	2	0	-1.08	-0.77	64.68
CMC	4	192.65	4	1	0	-0.56	-0.50	41.57

The excellent biological screening results against selected bacteria organisms and the high inhibition of DNA gyrase activity demonstrated by the new compounds CTC, CPC, CMC, including their good pharmacokinetic profile especially their low molecular weight recommend them as viable hits for optimization towards the design of novel antibiotic drug(s).

### Binding Mode Prediction

**Fig 3:**
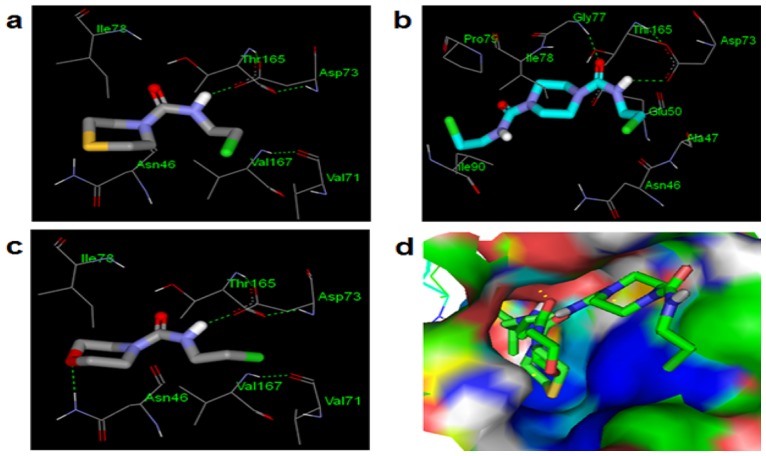
Binding conformations of the compounds (a) CTC, (b) CPC, (c) CMC and (d) is the solid surface representation of the protein binding site with all the three compounds in it. For subfigures a, b and c; the protein residues and ligands are in line and stick representations respectively. The atoms are in their standard colors for both the protein residues and ligands i.e. oxygen atom is in red color, chlorine atom in green color, hydrogen atom in white color, sulphur atom in yellow color etc. Polar contacts are shown in dash lines.

The poses of the three compounds within the active cavity of DNA gyrase was investigated to understand the essential intermolecular interactions existing between the protein-ligand complexes and employ the knowledge as a guide in structural optimization process of the potential drug molecules. The best docked poses of the compounds showed the key roles of the carbonyl (C = O) and secondary amino (R_2_NH) groups of the compounds because all the three compounds were found to use them in making two hydrogen bonds with the Thr165 hydroxyl group and Asp73 carboxylate group of the DNA gyrase respectively (Fig 3). The hetero sulphur and oxygen atoms in CTC and CMC respectively were used to establish hydrogen bonding with the Asn46 NH_2_ group (Fig 3a and 3c) but this interaction was not found in CPC, probably due to steric hindrance. It was rather observed that CPC related more with the protein residues through its extra substituted amide group which may have accounted for its very high affinity for the DNA gyrase. The binding conformation of CPC allowed the C = O and NH groups in the second substituted amide group to hydrogen bond with the Ile78 NH and Pro79 hetero-nitrogen atom of the protein groove (Fig 3b). Whereas, the terminal chlorine atoms found in CTC and CMC made hydrogen bond with Val167 and 171 NH groups, the pose of CPC allowed it to establish similar interaction with different residues (Ala47 and Asn46).

**Fig 4:**
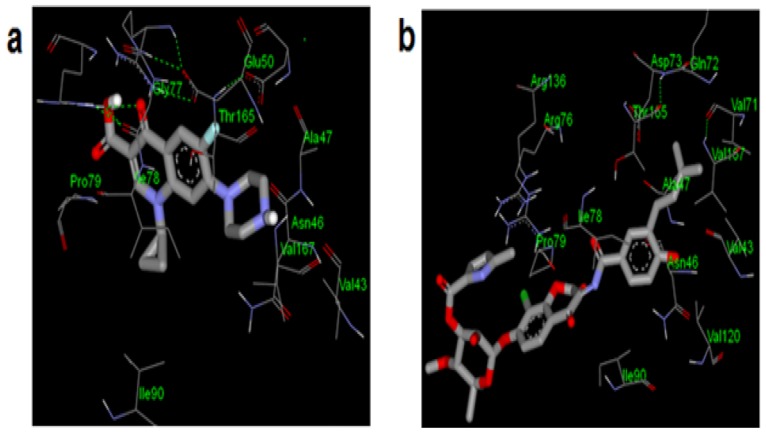
Binding conformations of the (a) native ligand (chlorobiocin) and (b) ciprofloxacin showing interactions with DNA gyrase active site residues similar to those observed in Fig 2a, 2b and 2c.

Analysis of binding configurations showed that the studied compounds (CTC, CPC and CMC) adopted similar binding modes to those of known inhibitors (Fig 4), hence, justifying the predicted binding modes.

## Conclusions

The antimicrobial profiles of the three 1-chloro-2-isocyanatoethane derivatives CTC, CPC and CMC against selected potential pathogens are presented in this study. The results highlight the prospect of the three compounds as future drug candidates; hence the need for further research in toxicity testing and overall *in vivo* studies. Besides, additional insight is needed in designing effective strategies to enhance trans-membrane partitioning of antibiotics particularly through the lipopolysaccharide ‘firewall’ of the Gram negative bacterial cell walls. The ability of the three compounds to inhibit activity of bacteria DNA gyrase was investigated by means of docking simulation and their possible binding mode predicted. The theoretical free energy of binding obtained from docking calculations showed that all the compounds performed better than chlorobiocin (cocrystallized ligand) and ciprofloxacin, in terms of ligand efficiency. The described binding mode identified key residues which could be targeted in rational structure optimization process. This approach could in the long run reduce the huge sums spent yearly on drug prospecting and development while at same time empowering antibiotics to save more lives through the acquisition of broad spectrum coverage. Presently, the only solution to the menace of antibiotic resistance lies in the discovery of new and more efficient drugs and the foundation is laid in the present study.
